# Relatives to Critically Ill Patients Have No Sense of Coherence: A Quality Improvement Article Using Mixed Methods

**DOI:** 10.1155/2016/6195894

**Published:** 2016-09-15

**Authors:** Jannie Laursen, Kristoffer Andresen, Jacob Rosenberg

**Affiliations:** Department of Surgery, Herlev Hospital, University of Copenhagen, Herlev Ringvej 75, 2730 Herlev, Denmark

## Abstract

*Aims and Objective*. To investigate the relatives' satisfaction and involvement on a general surgery ward regarding the critically ill patient.* Introduction*. Relatives to critically ill patients are affected both physically and mentally during the hospitalization of a family member. Research has shown that relatives do not always receive the attention they need from health professionals. There is a lack of studies that focus on relatives' satisfaction and involvement during their family members' hospitalization.* Design*. A mixed methods design was chosen.* Methods*. A quantitative study was conducted with 27 relatives to critically ill patients. All participated in a questionnaire and out of the 27 relatives, six participated in qualitative in-depth interviews.* Results.* The questionnaire revealed that relatives were dissatisfied with care and involvement. For further exploration of the dissatisfaction, a qualitative approach was used and the in-depth interviews revealed three themes: lack of continuity and structure, responsibility of coordination, and relatives feeling left on their own with no guiding and support.* Conclusion. *Health professionals' key role in relation to relatives must be guidance and support. Thereby, relatives can gain a sense of coherence during the hospitalization of a critically ill patient, which can lead to a greater satisfaction and thereby better support for the patient.

## 1. Introduction

Having a partner with a serious illness has an enormous impact on family life, and relatives are affected both physically and mentally [1]. Relatives living with a critically ill patient will often experience that their life is turned upside down, which can be very stressful. A study on relatives to patients with Alzheimer's disease showed that relatives were more distressed, showed poorer immune function, and had an increase in respiratory tract infections, compared to a control group [2]. The study found that even when the patient died, the relatives continued to show immunological downregulation for several years [2]. Family members are, in many cases, the main source of support for a critically ill patient and often hold valuable knowledge about the patient. Allowing relatives to be present and play a role in caring for the patient is often comforting for the patient and can improve care planning for the benefit of both patients and the hospital [3]. Research has shown that relatives do not always receive the attention they need from health professionals; the reason for this is often unclear [3]. Relatives can be seen as competent and important partners for health professionals. A study on frail and vulnerable patients indicated that quality of care improved in decision-making and exchange of knowledge was a collaboration between relatives and health professionals [4].

If care is not properly coordinated by the health professionals, the quality of care can be experienced as being inadequate and lead to frustration for patients and their relatives. A Canadian study showed that poor satisfaction from patients and relatives was due to health professionals' inability to coordinate care. The main source of dissatisfaction was rotation between health professionals, difficulties in making decisions, and lack of coordination between health professionals [5].

It has been shown that relatives receiving information, advice, and emotional support are more likely to be satisfied [6]. When caring for critically ill patients, health professionals must be aware of their role and the asymmetry this relationship contains. Being in a situation where a patient's life situation is changing will often lead to changes in the relatives' situation. Relatives will often experience an increase in pressure due to the hospitalization of their loved one, which can leave them with a feeling of hopelessness and despair. Hope is essential, because it enables people to cope with difficult situations and life changes [7]. Health professionals must be aware of the responsibility they have to influence the feeling of hope. It has been shown that health professionals did not have the knowledge of what relatives go through during the hospitalization of their partners [8, 9]. There are often no standardized protocols for including relatives in the care provided by health professionals in a general ward, and there is a great variation in the care offered for relatives within the hospitals [4].

The sooner the health professionals prepare interventions that include relatives in the care and treatment of the patient, the sooner they can prevent a later negative reaction from the relatives [10]. A recent study reported that relatives should be considered important in the treatment and care with patients [11]. This study measured the impact of different levels of social support on patients' self-care. Patients with a high level of social support found that having a supporting partner had a significant influence on several parameters, such as taking medications, managing fluid intake, and consulting health professionals [11]. Therefore, it is necessary to be aware of what relatives go through and ideally have a way of monitoring relatives' experience of quality of care [10]. A recent study reported that relatives should be considered important in the treatment and care with patients [11]. This study measured the impact of different levels of social support on patients' self-care. Patients with a high level of social support found that having a supporting partner had a significant influence on several parameters, such as taking medications, managing fluid intake, and consulting health professionals [11]. Therefore, it is necessary to be aware of what relatives go through.

The aim of this study was to investigate the relatives' satisfaction and involvement on a general surgery ward, regarding the critically ill patient.

## 2. Methods

This study was a mixed methods study designed to identify the relatives' involvement, satisfaction, and needs during hospitalization of a patient that became acutely ill. Data were collected in two steps, first a survey and then a study using in-depth interviews.

The reason for choosing a mixed method was because of its capacity to strengthen and explore the results found in one research approach compared to the other. In this case, it allowed the quantitative results from the survey to be explained with a qualitative study through in-depth interviews [12].

### 2.1. Participants

Inclusion criteria were relatives to patients with a deterioration in their illness within the last 48 hours defined as fulfilling one of the following criteria: an early warnings score ≥ 7 [13], having the mobile emergency team called for a consultation or transfer to the intensive care unit or another room for closer observation. Exclusion criteria were relatives with psychiatric disorder, language difficulties, or withdrawal of consent.

All participants were recruited from the surgical ward between March 1, 2014, and July 1, 2014.

### 2.2. Phase One: Questionnaire

The questionnaire used for the survey was a translated version of the nursing care survey 2002 NOSA [14]. The questionnaire has 12 items that illuminate the satisfaction of relatives. The questionnaire is structured so a score can be obtained from each item [14]. For this study, two items were excluded from the questionnaire, because they were regarding discharge, a theme not relevant for this study, since patients were not discharged at the time of data collection.

### 2.3. Scientific Considerations

The process of translation was aligned to the recommendations for translation and cultural adaption of surveys [15]. Approval to translate the nursing care survey 2002 NOSA [14] was obtained from the inventors.

### 2.4. Translation

A panel of three expert researchers translated each the nursing care survey 2002 NOSA from English to Danish. The three translations were then evaluated with discussion between the three researchers. When agreement was reached, the survey was translated back to English by a native speaking Dane. When the questionnaire was backward translated to English it was sent to the inventor in Australia for validation. When the translated questionnaire was sent back from Australia with comments, the Danish version was corrected and deemed ready for testing.

### 2.5. Validation of the Questionnaire

For further validation, the questionnaire was evaluated through individual interviews with relatives. Each participant was interviewed about the meaning of each question in the questionnaire in order to clarify misconceptions. A written conclusion was signed by each participant, after each interview, and used in the further validation process.

### 2.6. Analysis and Statistics of Quantitative Data

Each item could be responded on a 5-point Likert scale with 0 indicating total agreement and 4 indicating total disagreement with the statement (Table 1). A total score for each participant was calculated and converted to a score ranging from 0 to 100 by total score/max score × 100. Higher score indicating lesser agreement with statement/item. Responses for all participants for each item are presented as median and range. Furthermore, responses were analyzed with the use of cut-off values with the percentages of respondents in the categories “always” and “mostly.” The categories were >80% very good standard, 70–80% acceptable standard, and <70% requiring attention [14].

Comparison of total score for interview participants versus noninterview participants was performed with the Mann-Whitney *U* test. All analyses were done using SPSS version 22 (SPSS Inc., Armonk, NY: IBM Corp.).

### 2.7. Phase Two: In-Depth Interviews

For the in-depth interviews, the researchers were focusing on a circular motion, where the researcher was concerned with the relatives' experiences as they were lived. Here, the process becomes a dialogical method, where the researcher and the phenomenon being studied are combined together [16]. The in-depth interviews were preferred, as they made participants feel more comfortable to talk about their personal experiences, when they were done face to face as opposed to focus group discussions. This approach makes it more likely for the researcher to get in depth with the themes of a more sensitive character [17]. A semistructured flexible interview guide was developed from the themes in the questionnaire and then combined with findings from conversations with two relatives to patients that had become critically ill. This was done to secure that the responses contributed to a deeper understanding of the items in the questionnaire. The interview guide was validated by the first author's professional experiences. The first author performed the interviews, which took between 30 and 45 min.

### 2.8. Data Analysis

Each interview was recorded and then transcribed verbatim by an external contributor and carefully reviewed by Jannie Laursen and Kristoffer Andresen. The interviews were transcribed into full text and qualitative content analysis was used for analyzing the data [18]. The data were condensed and coded manually. The two researchers (Jannie Laursen, Kristoffer Andresen) performed the analysis in parallel processes; they subsequently discussed subthemes and themes which were then compared and reflected upon in an in-depth process.

### 2.9. Ethical Considerations

The study was approved by the Danish Data Protection Agency (Journal number 528-02971) and was exempt from ethical approval from The Ethical Committee of The Capital Region in Denmark, but a statement of exemption was obtained (Journal number H-2-2013-FSP56). Relatives who met eligibility criteria were identified by nurses at the surgical ward and asked by the first author whether they would participate in the study. The relatives were informed that they at any time could withdraw from the study, and it would not influence the patient's treatment or care. Before answering the questionnaire and/or participation in the interview, a consent form was signed and the relatives were reassured of the confidentiality both orally and in writing.

## 3. Results

We approached 50 potential relatives of whom 27 participated in the questionnaire, and six of them participated in in-depth interviews. Of the 27 participants, 11 were men. Age was between 28 and 80 years. All participants were family members, including parents, siblings, children, and spouses. Reason for declined participation for the 23 was due to stress, fatigue, or the loss of their loved ones.

### 3.1. Findings from the Questionnaire

In the questionnaire, median (range) score for all participants were 30 (0–70). For seven items out of 10, less than 70% of the participants indicated that they were “always” or “mostly” agreeing with the statement. This is an indication that a third of the relatives were unsatisfied with these items and that they require attention (see Table 1). The highest degree of dissatisfaction was found for items four “Were you involved in decisions about the care of your relative as much as you felt you needed to be” and eight “Did the health professionals anticipate and meet the needs of your relative”, both with only 48% of patients responding “always/mostly.” In order to further explore these items, the in-depth interviews were conducted.

### 3.2. Findings from In-Depth Interviews

The findings were based on the narratives from six relatives, four women and two men which participated in the in-depths interviews. During the analyses of the data, three themes emerged; the first one was lack of continuity and structure, the second one was responsibility of coordination, and the third one was relatives left alone with no guiding and support. All relatives were physically involved in the care of the critically ill patient and they were all very emotional and sensitive about the critical situation. All relatives were experiencing feelings such as frustration, anxiety, and a profound struggle to understand the unfortunate situation, which lead to the themes described in the following.

### 3.3. Lack of Continuity and Structure

This theme was focused on the relatives' perceptions of lack of continuity and structure. The relatives experienced that when the condition of the patient deteriorated and the situation became severed, the thing they needed the most from the health professionals was structure and some form of continuity in the further plan, one relative explained it as follows:

*“There was a new doctor every day, there were 47 different names to remember, and who should I call (?!). All they did was to state her (the patient) diagnosis, and send a request for a supervising doctor. I believe we all know what her diagnosis was and that the doctors needed a consultation from another doctor,”*

Everything inside the relatives was an emotional chaos and they were clinging to every little thing in the hope of gaining some sense of the situation. They needed continuity and structure to create a sense of coherence. When the relatives were not offered reconciliation from the health professionals, it often resulted in confusion and frustration, as a relative noted:

* “My nerves were frayed, the health professionals promised to take care of my husband, to check on him, but that just didn't happen”.*

In line with quantitative findings, only 59% of the relatives felt that the care was well organized. Often relatives felt that the health professionals were not able to create continuity in the care of, or during the admission of, the critically ill patients. The relatives felt that availability of information was poor and always at the request of them. Even though they were present and tried to be involved, the health professionals made no effort to include them in any way. This was commented on as follows:* “The health professionals saw me every day, so they knew who I was, but there was no attempt to include me and they knew that information was very important for us, that I knew what was going on, because my mom didn't always understand what was happening”*. Often the health professionals were vague in their attempts to meet the relatives and often the waiting time felt very long and uncertain, which only made it more urgent for the relatives to gain some kind of information and not have to bear all the responsibility on their own.

### 3.4. Responsibility of Coordination

The second theme was responsibility of coordination. The relatives felt that they had to take charge on all follow-ups regarding care and treatment; they felt that there were a lack of leadership and consistency presented by the health professionals. The lack of consistency and follow-up from health professionals often resulted in negative emotional reactions, which lead to some relatives having difficulties pursuing with their daily lives. It was by one relative commented on as

*“It is terrible for you as a spouse, to see your wife lying there, horrible when you can't see any progress and you feel the health professionals just talk and talk, but nothing happens. You can only interpret this as carelessness, as they don't take any responsibility, which leaves you feeling powerless”. *

They experienced that the health professionals were apathetic and indifferent, which often lead to difficulties in managing and coping with the given situation for the relatives:

*“I never received any explanation why they went in a new direction with my husband, which meant a lot of stress for me”.*

Consistent with the findings from the quantitative analysis, only 55% of relatives were answered by the health professionals in a way they could understand. This led to uncertainties in the situations, which often led to stress and anxiety for the relatives. Often the feeling of not being able to deal with the responsibility and not being offered any support or guiding from the health professionals led to relatives not having time to cope with their own emotions:

*“There was too much focus on coordinating things, making sure that all was running smoothly. If I shouldn't have done that, I could have been there for her, been more present and less stressed. I was very scared of what was going to happen and what I feared the most did happen”. *

Often when relatives did try and manage the increased responsibility and the lack of commitment from the health professionals, they felt that they were not given any authority to do so. The increase in responsibility and the severity of the situation only made them more desperate and resigned. In some cases, that meant that some of the relatives had a strong need to stay in control and take all the responsibility for care and treatment. They became more in need of control. A relative expressed it as* “I sit all day in my taxi, thinking, ‘should I call the hospital', hoping they had remembered to check on my mother. But then again it would be stupid, that will only signal that I didn't feel comfortable or relied on the system. But I do feel it's a madhouse, most of the time”*.

The division of roles and responsibilities between health professionals and relatives was not clear for them, as described by one relative:

*“It's not me who has to walk around and organize their work. It seems wrong”.*

Relatives felt that they had to be responsible and stay in control over the situation. If they did not, the critically ill patient would not get the correct or proper care. At the same time, the relatives felt anxious and confused and the weight from the responsibility was almost unbearable for them, as one relative indicated:

* “There is no consistency in anything, always swopping around, new doctors, and new nurses. No one takes any responsibility”.*

Many relatives felt that they had to be present most of the day, to make sure that the health professionals were offering the critically ill patient the needed care, according to one relative:

* “There were many situations where my mother did not understand what was happening if I wasn't there”*

Relatives felt that they had the responsibility to coordinate and secure that things were running smoothly. If they did not take part in caring for the critically ill patient and were physically and mentally present with a constant awareness of the situation, everything would collapse and everything would fall apart; it was described as follows:

* “I was told that she was hospitalized, but my dad said that I shouldn't hurry to the hospital, so I came here at one o'clock. I found her in her own dirt, she had urinated in her pants and I was indignant. It was like there had not been anyone to check on her. I was angry, and that's why I come early every day. I am checking that this is not happening again, I have to stay in control. It made me angry and sad”.*

The relatives had to manage care and treatment for the critically ill patient, to make sure that all was done professionally, even though they were not health professionals:

*“If I didn't take the leadership no one took responsibility”. *

Relatives felt they were not allowed to be relatives. It was not possible for them just being a relative. There was no room for that. They felt that the situation called for more professionalism than the health professionals delivered, which meant they had to be responsible for it. It was often described as

*“I didn't understand why I should attain work that was not mine. It was hard, that I had to play such a big part in coordinating things”.*

It was clear during the interviews that the responsibility to harmonize, the lack of structure, and the unclear division of roles left the relatives with a feeling of lack of coherence, which often lead them to a feeling of losing control, frustration, and mistrust of the system.

### 3.5. Relatives Felt Left Alone with No Guiding and Support

The third theme focused on health professionals' insufficiency in guiding the relatives. Many relatives were overwhelmed by the situation, when their loved one became critically ill. They felt that they were being left on their own and at the same time expected to maintain control; one relative indicated that

*“The role of just being relatives was hard to keep - not being able to come for a visit and just give her a hug was hard”.*

In line with quantitative findings, only 48% felt that the health professionals anticipated and met the needs of their relatives. Many relatives felt they had to advocate for the patient. They felt that they had to oversee the work of the health professionals in order to make sure that the patient received sufficient care. They had a direct responsibility for the patient, but at the same time they were torn between trusting the system and experiencing it as collapsing. One of the relatives described it as follows:

*“No explanation. I was just excluded. They simply said, that we have to let certain things go, which for me meant, that I had to take responsibility for my mother's care. I had to stay in control, because what if they missed something”. *

Many relatives felt that they had to use their full mental and emotional capacity to maintain some control over the situation. The communication with the health professionals was challenged by the feeling that they did not listen to them and were not taking charge over the situation. The amount of energy the relatives had to mobilize to be in control often exceeded what was possible. The relatives experienced a lack of care or concern from the health professionals, which was perceived as hard and unsatisfying for them. Relatives felt isolated in the situation, which left them with a feeling that no one understood what was going on. They felt detached from the situation and they did not feel that the health professionals were interested in them or their resources. Relatives were not an integrated part of the care for the critically ill patient and more perceived as someone who toke resources from the health professionals. One relative expressed it as* “I didn't receive any care as a spouse. I would have liked if they had talked to me, included me in their plans, spent a little time on me, and showed they empathized”*. Another relative stated,* “The culture is very much ‘them and us'. The health professionals decide that you have no claim as relatives”.*

When health professionals were not able to include and inform the relatives, it left the relatives feeling powerless. Often relatives felt they had to be the coordinator and secure that everything was running smoothly. Relatives' negative experiences would often damage their trust in the health care system.

## 4. Discussion

In this study, the aim was to investigate the relatives' satisfaction and involvement on a general surgery ward regarding the critically ill patient. The study investigated the level of information, the involvement of the relatives in the patient care, and the relatives' perspectives of quality of care, regarding the patients' needs, and health professionals' communication. The questionnaire results showed that a third of the participants did not seem satisfied with the quality of care. The qualitative results showed that in critical situations health professionals need to be present, caring, and empathic. This was not the general experience by the relatives as revealed by the responses to the questionnaire. Often they felt they were on their own, not skilled to cope with the situation, and in need of the health professional that could lead care and treatment and at the same time include them in the process.

The use of satisfaction as a tool to measure quality of care has previously been discussed although it is a common and often used measurement [19–21]. Earlier studies using satisfaction as a measurement found that being a woman, having a high educational level, or having a health professional's background often was associated with a decrease in satisfaction [4, 21]. However, these uncertainties were taken into account in the in-depth interviews, so that participants were of both sexes, there was a mixture of educational levels, and there were no health professionals among the relatives. A study on collaboration between relatives of elderly patients and nurses stated that relatives that were new in the role as relatives could also be more critical towards the healthcare system than others [4]. This is of course a factor to consider, as in this study there were relatives that were both new in the role and more experienced.

A third of the relatives stated that they were dissatisfied with the health professionals on the surgical ward. They were especially dissatisfied with the organization of the care and the involvement about the care. These results were consistent with other studies, showing that dissatisfaction often was associated with how the relatives were informed and was often associated with severity of the patient's illness [4, 22]. If patients were managed on a special unit and not a general ward, it was often seen that it had a positive influence on the level of satisfaction [4, 11, 22].

This study showed that relatives felt there were no structures during the admission and often they felt that they were left in the dark, with no ability to obtain information and with no consistency. These findings align with previous research, where relatives experienced both system and patient frustration [5, 6]. The frustration was often due to poor communication and long waiting time especially for feedback but also decisions about care. The study found that relatives often felt that the health professionals did not care and that they were met with negligence [5, 9]. These findings add to the evidence that relatives want to participate in decision-making and be treated as collaborators when the patient becomes critically ill.

Many of the challenges identified by the relatives were interrelated and often exacerbated each other. For example, a poor understanding on who was responsible for what led to a feeling of lack in feedback which increased the uncertainty further. Frustration with managing the proper care was aggravated by the health professionals' inability to coordinate and take charge and thereby not providing the appropriate comfort and security for the relatives. Better communication and improving the management of decision-taking were a key item to be able to guide the relatives in a more satisfactory way. Relatives expressed a need for better access to and clearer and more consistent feedback. A study on frustration expressed by relatives stated that they wanted face to face consultation, but because this is not always possible an alternative could be to use an electronic consulting system as a supplement to the face to face communication [5].

A more holistic approach is obviously needed. Here, the relatives would be considered as important collaborators to the critically ill patient, providing relevant and significant information and at the same time maintaining energy to be supporting and caring for the critically ill patient. A study on spouses' needs for professional support showed that many spouses felt that the health professionals were annoyed by their presence and that the spouses often were met by indifference [9]. When people experienced lack of coherence due to a life changing event, it was often shown in difficulties in managing and coping with even the smallest things. A hospital culture that provides support and consistence will often lead to a more manageable situation. If the culture values the role of people, it will be perceived as more sustaining and leave a feeling of meaningfulness, which can help support relatives in a more manageable way [23]. Relatives can be perceived as both resources and potential clients and the stress they experience can affect their health on a long term [1]. Being a relative to a critically ill patient will often lead to the loss of control. The feeling of loss of control will often collide with the need to see the world in a clear, ordered, and structured way [24]:

*“There is a need to also study the perception of care for relatives from the health professionals' point of view. There is probably a range of reasons why the health professionals do not provide the care for relatives that they so desperately need, and it is important to characterize the health professionals' experience in order to make meaningful interventions for the improvement of the care for relatives. However, it was not within the scope of this study to also cover the experience of the health professionals.”*

## 5. Strengths and Limitations of the Study

Combining results from two different research approaches may be a challenge. However, by interviewing six participants in the in-depth interviews, we exemplified what the relatives dissatisfaction covered, which can lead to new interventions towards including relatives in general wards. The study had a relatively small sample size. The sample consisted only of relatives to patients that became acutely ill. The reason for this was that very little research has been done in the field of a general surgical ward with patients that suddenly became acutely ill. These patients' relatives take a lot of focus due to the fact that their situation unexpectedly changes.

## 6. Conclusion

When patients acutely become critically ill, it often affects the whole family. A third of the participants in this study were unsatisfied with the organization and the involvement in care. The findings implied that they were left with no guiding or support from the health professionals, which often led to the feeling of loss of control. Health professionals' key role in relation to relatives must be guidance and support. Thereby, relatives can gain a sense of coherence during the hospitalization of a critically ill patient, which can lead to a greater satisfaction and thereby better support for the patient.

## Figures and Tables

**Table 1:** The 10 statements from the questionnaire, each with the percentage of respondents indicating that they were *always* or *almost always* agreeing to the statement. A percentage lower than 70% is an indication that improvement is needed [14].

Questions	Always/mostly (%)
Was the nursing staff that took care of your relative present when you needed them?	55
Were the nurses accommodating and easy to talk to?	78
Did the health professionals maintain confidentiality while caring for your relative?	70
Were you involved in decisions about the care of your relative as much as you felt you needed to be?	48
Did the health professionals explain why the care was given in a way which was understandable for you?	67
Was the nursing staff responsive to the special needs and concerns of your relative?	55
Did you feel that the care of your relatives was well organized?	59
Did the health professionals anticipate and meet the needs of your relative?	48
When you asked questions to the health professionals did they answer in a way you could understand?	55
Were you involved in planning the discharge of your relative from the department?	78
